# VEGF in the Crosstalk between Human Adipocytes and Smooth Muscle Cells: Depot-Specific Release from Visceral and Perivascular Adipose Tissue

**DOI:** 10.1155/2013/982458

**Published:** 2013-07-09

**Authors:** Raphaela Schlich, Miriam Willems, Sabrina Greulich, Florian Ruppe, Wolfram Trudo Knoefel, D. Margriet Ouwens, Bujar Maxhera, Artur Lichtenberg, Jürgen Eckel, Henrike Sell

**Affiliations:** ^1^Paul-Langerhans-Group, Integrative Physiology, German Diabetes Center, Auf'm Hennekamp 65, 40225 Düsseldorf, Germany; ^2^Department of General-, Visceral-, and Pediatric Surgery, Heinrich-Heine-University and University Hospital Düsseldorf, 40225 Düsseldorf, Germany; ^3^Signal Transduction, Institute of Clinical Biochemistry and Pathobiochemistry, German Diabetes Center, 40225 Düsseldorf, Germany; ^4^Department of Endocrinology, Ghent University Hospital, 9000 Ghent, Belgium; ^5^Department of Cardiovascular Surgery, Düsseldorf University Hospital, 40225 Düsseldorf, Germany

## Abstract

Adipose tissue secrets adipokines and fatty acids, which may contribute to obesity-associated vascular dysfunction and cardiovascular risk. This study investigated which factors are responsible for the synergistic effect of adipokine and oleic acid- (OA-) induced proliferation of human vascular smooth muscle cells (VSMC). Adipocyte-conditioned medium (CM) from human adipocytes induces proliferation of VSMC in correlation to its vascular endothelial growth factor (VEGF) content. CM increases VEGF-receptor (VEGF-R) 1 and 2 expression and VEGF secretion of VSMC, while OA only stimulates VEGF secretion. VEGF neutralization abrogates CM- and OA-induced proliferation and considerably reduces proliferation induced by CM and OA in combination. VEGF release is higher from visceral adipose tissue (VAT) of obese subjects compared to subcutaneous adipose tissue (SAT) and VAT from lean controls. Furthermore, VEGF release from VAT correlates with its proliferative effect. Perivascular adipose tissue (PAT) from type 2 diabetic patients releases significantly higher amounts of VEGF and induces stronger proliferation of VSMC as compared to SAT and SAT/PAT of nondiabetics. In conclusion, VEGF is mediating CM-induced proliferation of VSMC. As this adipokine is released in high amounts from VAT of obese patients and PAT of diabetic patients, VEGF might link adipose tissue inflammation to increased VSMC proliferation.

## 1. Introduction

Obesity has become a major worldwide health problem, especially in industrial countries, and is associated with a number of metabolic diseases and secondary complications, including insulin resistance, type 2 diabetes, atherosclerosis, and cardiovascular disease [1]. It is well established that adipose tissue is an endocrine organ releasing lipid mediators and a variety of proteins, the so-called adipokines [2]. Increasing evidence indicates that obesity is causally linked to a chronic low-grade systemic inflammatory state [3, 4] that contributes to obesity-associated vascular dysfunction and cardiovascular risk [5]. Obesity is strongly related to the development of atherosclerosis in humans as well as in various animal models [6, 7]. In this context, visceral obesity confers the highest risk for the development of metabolic and cardiovascular diseases [8]. Specifically in the pathophysiology of vascular diseases, perivascular adipose tissue might play an important role because almost all blood vessels are surrounded by this fat depot, and due to the fact that perivascular adipocytes are not separated from the blood vessel wall by an anatomic barrier, the secretion of adipokines by this fat depot may provide a new link between obesity and vascular complications [9]. However, till now the mechanism how visceral and perivascular fat enhances the risk of metabolic and cardiovascular disease is not completely unraveled. Besides endothelial cells, vascular smooth muscle cells (VSMC) represent one of the major cell types of the vascular wall retaining homeostasis of the vessel wall. Arterial wall thickening is mediated by migration of VSMC from the media to intima and their concomitant proliferation. We previously established a model of adipocyte-conditioned medium (CM) inducing VSMC proliferation [10]. The combination of CM with oleic acid (OA) increased the proliferation in a synergistic way via induction of iNOS expression, NO production, and proinflammatory signaling. However, until now the mechanisms and factors, which are responsible for aberrant VSMC proliferation induced by lipid mediators and adipokines, are not fully understood. Therefore, the main objective of this study was to identify potential candidates that mediate the crosstalk between adipose tissue and VSMC potentially linking obesity and atherosclerosis. Using paired biopsies of visceral and subcutaneous adipose tissue (VAT and SAT) as well as perivascular adipose tissue (PAT) derived from lean, obese, and obese type 2 diabetic patients, this study is also aimed at relating the *in vitro* findings to a more physiological setting. Our study demonstrates that the vascular endothelial growth factor (VEGF) content of CM is positively correlated with CM-induced VSMC proliferation and that VEGF neutralization completely abrogates CM- and OA-induced proliferation. CM and VEGF regulate expression of VEGF-receptor (VEGF-R) 1 and 2. The secretory output from VAT and PAT of patients with type 2 diabetes and/or obesity contains high amounts of VEGF and stimulates proliferation and VEGF-R1/2 expression as compared to SAT.

## 2. Materials and Methods

### 2.1. Materials

Reagents for SDS-PAGE were supplied by Amersham Pharmacia Biotech (Braunschweig, Germany) and by Sigma (Munich, Germany). Polyclonal antibodies raised against VEGF-R 1 and 2 were supplied by cell signalling technology (Frankfurt, Germany), and the anti-actin antibody came from Abcam (Cambridge, Great Britain). Vascular endothelial growth factor (VEGF) neutralization was achieved by pretreatment of CM with 1 *μ*g/mL VEGF-neutralizing antibody from R&D Systems (Wiesbaden-Nordenstadt, Germany) for 1 hour. HRP-conjugated goat anti-rabbit and goat anti-mouse IgG antibodies came from Promega (Mannheim, Germany). Collagenase NB4 was obtained from Serva (Heidelberg, Germany). Troglitazone and BSA (fraction V, fatty acid free, low endotoxin) were obtained from Sigma (München, Germany). Human recombinant VEGF was purchased from Millipore GmbH (Schwalbach, Germany). The cell proliferation ELISA (BrdU, chemiluminescent) and protease inhibitor cocktail tablets were from Roche (Mannheim, Germany). VEGF ELISA kits were purchased from BioVendor GmbH (Heidelberg, Germany). FCS was supplied by Gibco (Invitrogen, Carlsbad, USA). Sodium salt of OA (Sigma, München, Germany) was dissolved in water as a 6 mM stock solution and was further diluted in sterile serum-free VSMC medium containing 4% (wt/v) BSA for coupling. OA was applied to VSMC at a final concentration of 100 *μ*mol/L for 18 h. All controls of experiments involving fatty acids were treated with BSA alone. All other chemicals were of the highest analytical grade commercially available and were purchased from Sigma. 

### 2.2. Culture of Human Adipocytes and CM Generation

**Figure 1:**
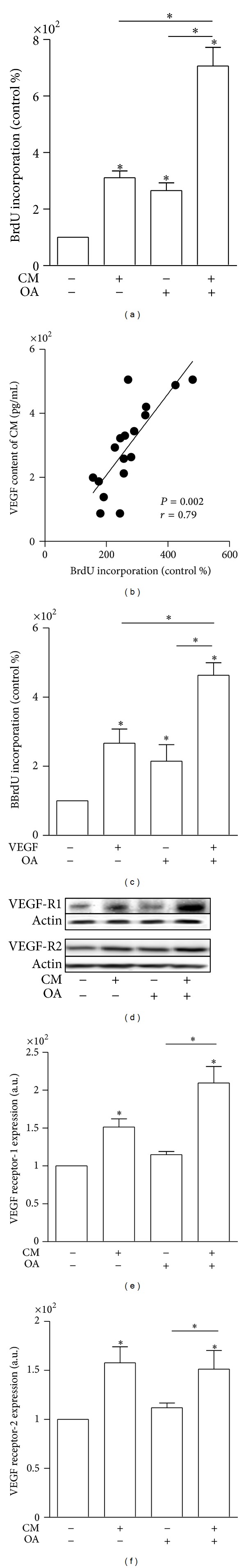
CM-induced proliferation is mediated by VEGF. (a) VSMC were serum starved for 24 h and subsequently incubated with CM, 100 *μ*mol/L OA, or the combination of CM and OA in the presence of BrdU for 18 h. Incorporation of BrdU into DNA was measured. Data are expressed relative to control, taken as 100%. Data are mean values ± SEM of three independent experiments. *P < 0.05 compared to control or designated treatment. (b) The proliferative effect of CM correlates significantly with its VEGF content as measured by ELISA. Statistical result of linear regression analysis is indicated in the graph. (c) VSMC were serum starved for 24 h and subsequently incubated with 250 pg/mL VEGF, 100 *μ*mol/L OA, or the combination of VEGF and OA in the presence of BrdU for 18 h. Incorporation of BrdU into DNA was measured. Data are expressed relative to control, taken as 100%. Data are mean values ± SEM of three independent experiments. *P < 0.05 compared to control or designated treatment. (d–f) VSMC were treated with CM, OA, and their combination for 24 h. Total cell lysates were resolved by SDS-PAGE and immunoblotted with specific VEGF-R1 and -2 antibodies. A representative blot is shown. Data are mean values ± SEM of three independent experiments. All data were normalized to the level of actin expression and are expressed relative to the control.

**Figure 2:**
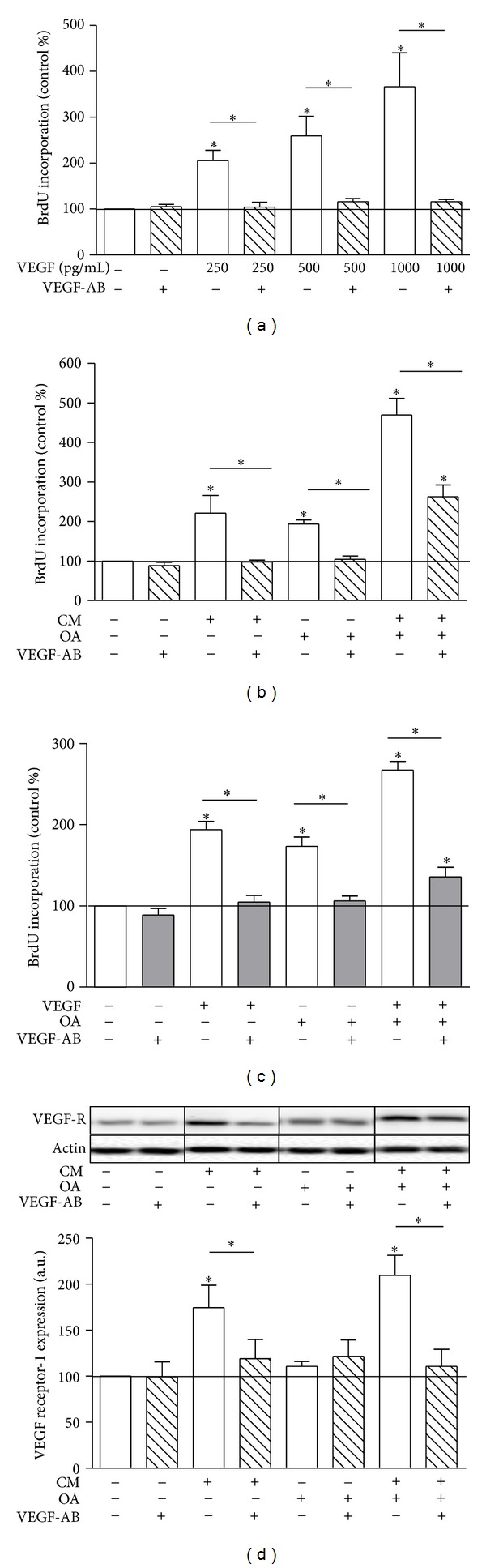
VEGF neutralization prevents CM- and OA-induced proliferation. Cells were treated with 250, 500, and 1000 pg/mL VEGF (a), CM, OA and CMOA (b and d), and accordingly VEGF, OA, and VEGFOA (c) as described in the legend to Figure 1 in the presence or absence of a specific neutralizing VEGF antibody for 24 h. (a–c) BrdU incorporation into DNA was determined as described in the legend to Figure 1. Data are expressed relative to control. (d) Total cell lysates were resolved by SDS-PAGE and immunoblotted with a specific VEGF-R1 antibody. For VEGF-R1, lanes were excised from a single western blot and displayed in the present order. Data are mean values ± SEM of three independent experiments. All data were normalized to the level of actin expression and are expressed relative to the control. *P < 0.05 compared to untreated control or designated data.

Subcutaneous adipose tissue was obtained from healthy lean or moderately overweight women (*n* = 23, body mass index (BMI) 26.1 ± 1.1, and aged 36.6 ± 2.0 years) undergoing plastic surgery. The procedure was approved by the ethical committee of the Heinrich-Heine-University (Düsseldorf, Germany). All subjects were healthy and free of medication and had no evidence of metabolic diseases according to routine laboratory tests. Preadipocytes were isolated by collagenase digestion of adipose tissue as previously described by us [11]. Seeded preadipocytes were induced to differentiation into adipocytes over 15 days as previously described by us [10]. The degree of differentiation was determined by oil red staining and induction of adiponectin. Differentiated adipocytes were used for the generation of adipocyte-CM, as recently described by us [12]. Briefly, CM was generated by culturing adipocytes for 48 h in VSMC basal medium (PromoCell) with addition of 50 ng/mL amphotericin b and 50 *μ*g/mL gentamycin. Each CM was tested for its proliferative effect and the content of adiponectin (negatively correlated to proliferation) [10]. A more-detailed characterization of CM was described previously by us [12, 13]. CM generated from *in vitro* differentiated adipocytes was used for all experiments presented in Figures 1 and 2.

### 2.3. Generation of CM from Explants of SAT, VAT, and PAT

**Figure 3:**
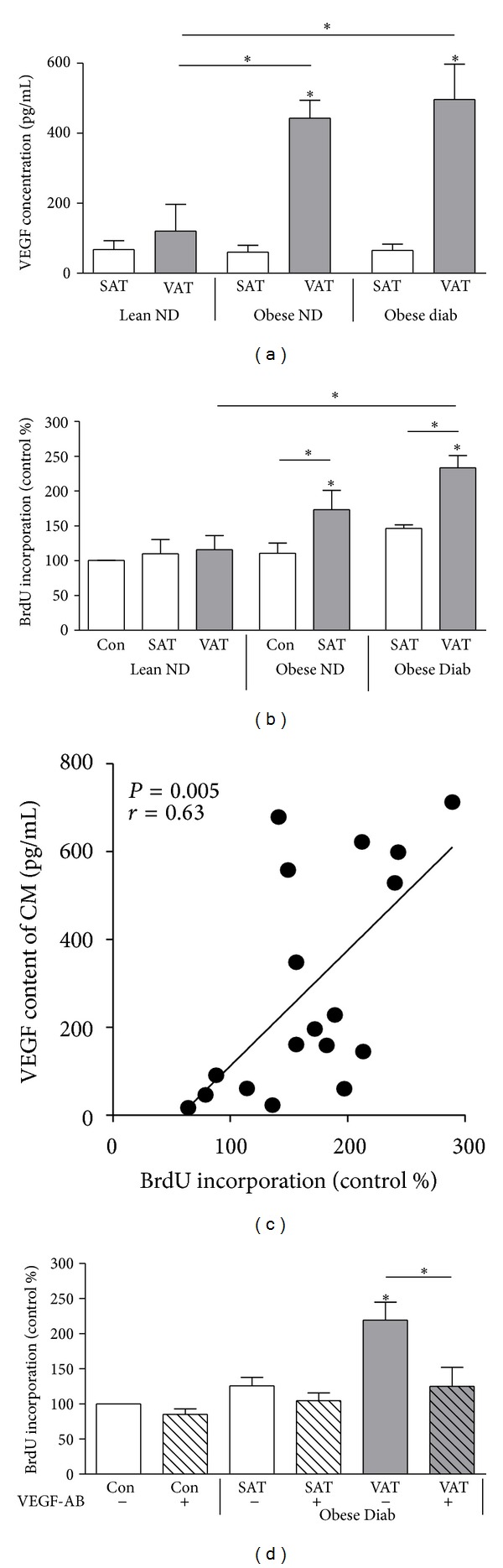
VEGF release is higher from VAT of obese subjects correlating with induction of VSMC proliferation. (a) VEGF content of CM from VAT and SAT was measured in duplicates by ELISA. (b and d) VSMC were treated with CM from paired SAT and VAT of lean nondiabetic (ND), obese ND, and obese type 2 diabetes (Diab) patients for 24 h. For (d), VSMC were treated in the presence or absence of a VEGF-neutralizing antibody. VSMC proliferation was determined by measuring the incorporation of BrdU into DNA. Data are expressed relative to the basal control value, which was set as 100%. Data are presented as mean ± SEM from five independent experiments. For (d), all data were normalized to the level of actin expression and are expressed relative to the control *P < 0.05 compared to control or designated data. For (d), lanes were excised from a single western blot and displayed in the presented order. (c) The proliferative effect of CM from VAT correlates significantly with its VEGF content as measured by ELISA. Statistical result of linear regression analysis is indicated in the graph.

**Figure 4:**
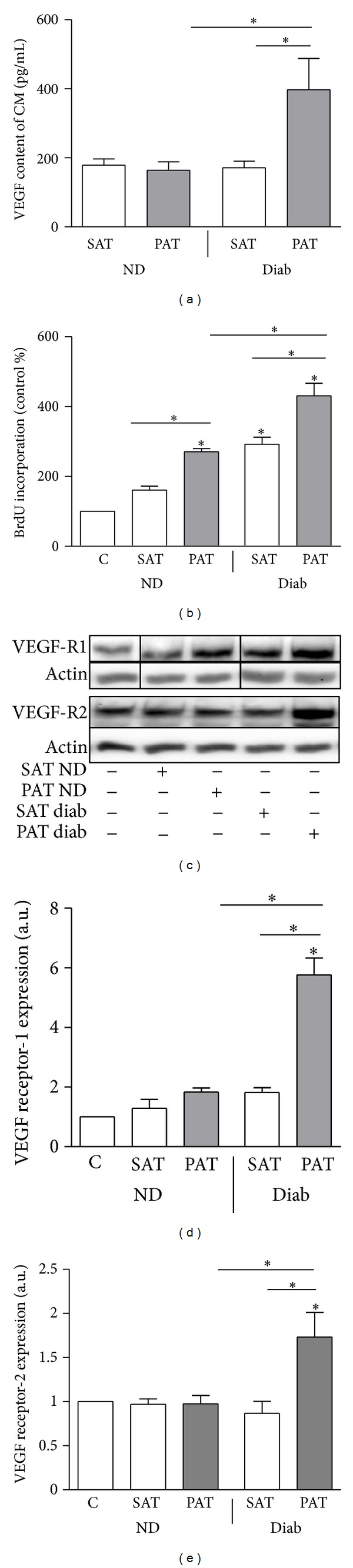
VEGF release is higher from PAT of type 2 diabetic patients correlating with VSMC proliferation and VEGF-R1/2 expression. (a) VEGF content of CM from PAT and SAT was measured in duplicates by ELISA. (b) VSMC were treated with CM from paired SAT and PAT of patients with type 2 diabetes (Diab) and nondiabetics controls (ND) for 24 h. The proliferation was determined by measuring the incorporation of BrdU into DNA. Data are expressed relative to the basal control value, which was set as 100%. Data are presented as mean ± SEM from five independent experiments. (c–e) Total cell lysates were resolved by SDS-PAGE and immunoblotted with specific VEGF-R1 and 2 antibodies. Data are mean values ± SEM of three to five independent experiments. All data were normalized to the level of actin expression and are expressed relative to the control. *P < 0.05 compared to control or designated data.

Paired biopsies from subcutaneous (SAT) und visceral adipose tissue (VAT) were obtained from lean patients without diabetes (*n* = 10, body mass index (BMI) of 21 ± 1 kg/m^2^ aged 67 ± 6), obese patients without diabetes (*n* = 11, BMI of 38 ± 3 kg/m^2^ aged 56 ± 6 years) and obese patients with type 2 diabetes (*n* = 6, BMI of 37 ± 6 kg/m^2^ aged 56 ± 5 years) undergoing elective surgery such as hernia, gall bladder surgery, and other noninflammatory and nonmalignant causes. Human perivascular (PAT) and SAT biopsies were obtained from patients with type 2 diabetes (*n* = 5, BMI 29 ± 7 kg/m^2^, aged 68 ± 9 years) and patients without type 2 diabetes (*n* = 9, BMI 28 ± 4 kg/m^2^, aged 69 ± 12 years) undergoing coronary artery bypass surgery. Information on the diabetes status was obtained from the medical records of the patients. PAT was collected from the coronary artery and used to generate CM as described [14, 15] using 100 mg of adipose tissue explant to generate 1 mL of CM. After 24 h, CM was collected and stored in aliquots at −80°C until further use. CM generated from SAT and VAT was used for all experiments presented in Figure 3, and CM generated from SAT and PAT was used for all experiments presented in Figure 4.

### 2.4. Culture of Human Vascular Smooth Muscle Cells (VSMC)

Primary human coronary artery VSMC were obtained from PromoCell (Heidelberg, Germany). VSMC from four different donors (Caucasian, male, 23, 31, and 40 years old; female, 56 years old) were used as subconfluent cells of passage 3. Cells were characterized as VSMC by morphologic criteria and by immunostaining with smooth muscle *α*-actin.

### 2.5. *In Vitro* Analysis of VSMC Proliferation

To monitor DNA synthesis, VSMC were seeded in 96-well culture dishes and allowed to attach for 24 h, followed by serum starvation for an additional 24 h period. Cells were then stimulated for 18 h as outlined above in the presence of BrdU (10 *μ*M). 10.000 VSMC per 15 mm² well were incubated with the CM of 35.000 adipocytes. The BrdU ELISA Kit was used to determine proliferation according to the manufacturer's protocol. Signals were visualized and evaluated on a LUMI Imager work station (Boehringer, Mannheim, Germany). 

### 2.6. Immunoblotting

VSMC were treated as indicated and lysed in a buffer containing 50 mM HEPES, pH 7.4, 1% TritonX100, complete protease inhibitor, and PhosStop phosphatase inhibitor cocktail. After incubation for 2 h at 4°C, the suspension was centrifuged at 10.000 ×g for 15 min. Thereafter, 5 *μ*g protein of lysates were separated by SDS-PAGE using 10% horizontal gels and transferred to polyvinylidene fluorid filters in a semidry blotting apparatus [16]. Filters were blocked with Tris-buffered saline, containing 0.1% Tween and 5% nonfat dry milk, and subsequently incubated overnight with a 1 : 1000 dilution of the appropriate antibodies. After washing, filters were incubated with secondary HRP-coupled antibody and processed for enhanced chemiluminescence detection using Immobilon HRP substrate (Millipore, Billerica, USA). Signals were visualized and evaluated on a VersaDoc work station (Bio-Rad Laboratories, Munich, Germany).

### 2.7. Presentation of Data and Statistics

Data are expressed as mean ± SEM. One-way ANOVA (post hoc test: Bonferroni's multiple comparison test) was used to determine statistical significance. All statistical analyses were done using Prism (GraphPad, La Jolla, USA) considering a *P* value of less than 0.05 as statistically significant. Corresponding significance levels are indicated in the figures.

## 3. Results

### 3.1. VEGF Content of CM Correlates with CM-Induced Proliferation

We have previously shown that CM from *in vitro* differentiated human adipocytes induces proliferation of human coronary artery VSMC with a large majority of the CMs tested inducing a prominent proliferation of more than 2-fold compared to control [10]. While CM and OA induce similarly strong proliferation of VSMC, their combination increased the proliferation in a synergistic way (Figure 1(a)), as reported previously [10]. The proliferative effect of CM negatively correlated with its adiponectin concentrations, but no correlation was found with IL-6 in CM [10]. Correlating CM content of growth inducing factors to CM-induced proliferation, we found that CM-induced proliferation strongly correlated with the VEGF content of the respective CM (Figure 1(b)). When replacing CM by VEGF at the average concentration found in the tested CM (250 pg/mL human recombinant VEGF), the proliferative effect of CM could be mimicked (Figure 1(c)) in accordance with previous data [10]. Treatment with CM, OA, or the combination of CMOA induced VEGF secretion of VSMC (control 178 ± 20 pg/mL, CM-treated 507 ± 20 pg/mL, OA-treated 414 ± 52 pg/mL, and CMOA-treated cells 1109 ± 69 pg/mL, *n* = 4) as previously described [10]. These data suggest that VSMC significantly contribute to proliferation by releasing VEGF for autocrine/paracrine stimulation. CM and the combination of CMOA also increased the expression of VEGF-R1 and -2, whereas OA had no effect on its expression (Figures 1(d)–1(f)).

### 3.2. VEGF Is an Important Factor for CM- and OA-Induced VSMC Proliferation

In order to assess the importance of VEGF in CM-induced proliferation, VEGF was neutralized with a specific antibody. To establish effective VEGF neutralization, VEGF was applied at concentrations ranging from 250 pg/mL to 1 *μ*g/mL, which mimics both the average VEGF concentration in CM and spans to the highest VEGF release by VSMC observed after CMOA treatment (Figure 2(a)). Neutralizing VEGF prevented the CM-, VEGF-, and OA-induced proliferation completely (Figures 2(b) and 2(c)), underlining our hypothesis that CM and OA increase VSMC proliferation via VEGF. VEGF blocking significantly reduced the proliferative effect of the combination CMOA, which is still elevated compared to untreated control (Figure 2(b)), illustrating that under this condition VEGF is not the only factor involved in the proliferative effect. Furthermore, the induction of VEGF-R1 by CM and the combination of CM and OA was also completely prevented by VEGF blocking (Figure 2(d)).

### 3.3. VEGF Release Is Higher from VAT of Obese Subjects Compared to SAT and VAT from Lean Controls Correlating with VSMC Proliferation

VEGF release from VAT was significantly higher compared to SAT in both obese subjects without type 2 diabetes and with type 2 diabetes (Figure 3(a)). On the other hand, VEGF release was low and comparable from VAT and SAT of lean subjects. Accordingly, VSMC proliferation was induced by CM from VAT of obese subjects, but not by CM from SAT, VAT from lean patients, and SAT from obese patients (Figure 3(b)). Similar to CM derived from human adipocytes (see Figure 1(b)), the VEGF content of CM from VAT correlated significantly with the proliferative effect of the respective CM (Figure 3(c)). However, VEGF content of CM from SAT was not correlated to proliferation (data not shown). Neutralization of VEGF prevented proliferation induced by CM from VAT of obese subjects with type 2 diabetes (Figure 3(d)). 

### 3.4. PAT of Type 2 Diabetic Patients Releases Increased Amounts of VEGF and Induces Significant Proliferation of VSMC

The release of VEGF was comparable from SAT and PAT of the nondiabetic subjects and from SAT of patients with type 2 diabetes (Figure 4(a)) while its release by PAT of type 2 diabetic patients was significantly increased. Accordingly, CM from PAT of type 2 diabetic patients induced the strongest proliferative effect on VSMC (Figure 4(b)). In both groups of patients, CM from PAT induced a stronger proliferation as compared to CM from SAT. Furthermore, CM from PAT of patients with type 2 diabetes induced a 2- to 4-fold increase of VEGF-R1 and -2 expression in VSMC (Figures 4(c) and 4(d)). In contrast, PAT of nondiabetics exerted a comparable effect to the respective SAT. Our findings suggest that VEGF may be an important adipokine produced in PAT especially from patients with type 2 diabetes that might directly induce proliferation and expression of VEGF-R1 in VSMC.

## 4. Discussion

Obesity is strongly related to the development of cardiovascular diseases, and adipokines have been suggested to be a molecular link in this relationship [4, 17]. This study was designed to elucidate mechanisms on how the secretory output from adipose tissue is related to VSMC proliferation. We could show in a previous study that CM of *in vitro* differentiated adipocytes induces proliferation of VSMC in negative correlation to the adiponectin content of CM [10]. Searching for an active component of CM being responsible for VSMC proliferation, we found VEGF in CM to be significantly correlated with proliferation. VEGF is traditionally known as an endothelial cell-specific growth factor, which modulates vascular disease by inducing endothelial proliferation mainly through the VEGF-R2 [18]. However, an increase of VEGF and VEGF-Rs could be observed in other injured cells of the arterial wall like monocytes and VSMC [19]. Vascular inflammation and the proliferation of endothelial cells as well as VSMC are enhanced through angiotensin II-induced VEGF release and expression of VEGF-R [20–23].

Here, we report that CM and OA as well as their combination induce release of VEGF also by VSMC, which in turn might autostimulate proliferation. This effect is similar to hypoxia-induced proliferation of VSMC, where an autocrine proliferative action of VEGF has been described [24]. In addition, it has been described that proinflammatory stimulation with angiotensin II and IL-1beta and activation of JNK induce VEGF release by VSMC [25, 26]. It might be speculated that proinflammatory adipokines present in CM are, therefore, responsible for the induction of VEGF. Furthermore, VEGF might autostimulate its release which might be worth to study in the future. In addition to stimulating the release of VEGF from VSMC, CM induced VEGF-R1 and -2 expression which mediate proliferation and migration of VSMC [27]. Our data suggest that both VEGF-Rs are regulated by VEGF itself, as neutralizing VEGF prevented CM-induced expression of these receptors in parallel to proliferation. In fact, a regulation of VEGF-R2 by VEGF has already been proposed [28]. Interestingly, OA stimulates VEGF secretion but not the expression of VEGF-Rs. As oleic acid has complex effects on VSMC, it can only be speculated that it directly or indirectly affects VEGF-R expression by still unknown mechanisms.

VEGF is a proinflammatory factor [29, 30], and we previously demonstrated that CM activates NF-*κ*B signaling that is essential for CM-induced proliferation [10]. VEGF is not primarily activating NF-*κ*B at concentrations measured and used in this study [29]. However, CM contains various adipokines including MCP-1 and chemerin [12, 31] that have been described to strongly activate NF-*κ*B. Activated NF-*κ*B could then in turn lead to increased VEGF expression and release as described in the literature [32]. Thereby, proinflammatory factors in CM may contribute to proliferation by inducing VEGF via NF-*κ*B. Blocking VEGF with a specific neutralizing VEGF-antibody reduced the CM, OA, and VEGF-induced proliferation of VSMC completely. In contrast, the strong proliferative effect of the combination of CM and OA was not totally prevented, illustrating that VEGF is not the only important factor for the synergism of CMOA. As CM contains various adipokines such as IL-6, IL-8, or MCP-1 [12], it is possible that some of these factors induce proliferation in addition to VEGF and that these factors might also be involved in the synergistic effects of CM and OA. Several adipokines such as leptin and resistin affected VSMC proliferation and function [33, 34] but are not probable to play a role in this scenario. Leptin release by adipocytes differentiated *in vitro* is too low to explain CM-induced proliferation [35], and resistin is only secreted from mouse but not from human adipocytes [12].

We show here that VEGF release is higher from VAT of obese subjects irrespective of their metabolic status. Previous studies have shown that VEGF expression is already increased along with increased inflammatory adipokines in VAT of children compared to SAT [36]. In adults, VEGF expression in obese patients is higher in VAT as compared to SAT [37]. Increased VEGF expression might be associated with higher inflammation in VAT as VEGF expression, and secretion is stimulated by inflammatory cytokines such as IL-6 [38]. Recently, it has also been suggested that hypoxia can induce VEGF expression in expanded adipose tissue [39]. VEGF in adipose tissue is recently discussed both as a “bad” and a “good” guy. One important function of VEGF within adipose tissue is to stimulate angiogenesis, which is crucial for increasing blood capillaries in expanding adipose tissue by stimulating endothelial cell growth. Here, VEGF is described as beneficial for preventing adipose tissue hypoxia and thus inflammation. In fact, several groups have demonstrated that inducible overexpression of VEGF in adipose tissue prevents obesity-induced hypoxia and inflammation in adipose tissue in parallel to improved metabolic phenotype [40–42]. Conversely, adipose-specific ablation of VEGF decreases vascularization and increases inflammation together with deteriorated metabolic control in high-fat diet-treated mice [42]. In the context of existing obesity, increased VEGF expression is rather seen as another stimulator of proinflammatory adipocytes further deteriorating adipose tissue function. Here, inducible ablation of VEGF in adipose tissue protects from obesity and induces brown adipose tissue within white adipose tissue in mice [43]. Taken together with another similar study using different mouse models [41], it has been suggested that the effects of VEGF within adipose are context dependent. While high VEGF expression in already expanded adipose tissue deteriorates metabolic control, increased angiogenic activity by VEGF induction during expansion of adipose tissue is beneficial to maintain normal metabolic function. As no detailed studies on VEGF expression of human adipose tissue during obesity development exist, further work is needed to expand this view to the human situation.

It has been proposed that PAT is involved in a paracrine crosstalk with cells of the vascular wall and that adipokines are major players in mechanisms linking PAT to inflammation and vascular dysfunction [44, 45]. Here, we demonstrate that PAT from patients with type 2 diabetes is characterized by a significantly higher VEGF release as compared to SAT and also compared to PAT from nondiabetics. Higher VEGF release is paralleled by a stronger induction of VEGF-R1 and -2. The use of paired biopsies from human SAT and PAT further strengthens the notion of VEGF release from PAT for a potential paracrine crosstalk. The VEGF content of CM from SAT and PAT from patients without diabetes as well as SAT from type 2 diabetic subjects was similar but did not have the same effect on VSMC proliferation. It should be noted that the use of adipose tissue explants is a more complex setting than using CM from adipocytes as the secretory output from adipose tissue explants also contains factors derived from other cell types present in adipose tissue as preadipocytes and macrophages. Further work will be needed to elucidate the differences in adipokine expression and secretion between PAT and other fat depots in health and disease in order to identify other putative factors responsible for PAT-induced vascular dysfunction.

A limitation of the study is that the small amounts of CM that could be prepared from the surgical biopsies from PAT disallowed experiments assessing the impact of VEGF neutralizing antibodies. In this respect, it should be noted that the secretory profile of human adipocytes and adipose tissue biopsies is complex, as illustrated for example, by disease and depot-specific differences in the adipokines released [15, 46]. The fact that VEGF content and the potential of PAT-derived conditioned media to induce VSMC proliferation do not seem to fully correlate suggests that a contribution of other adipokines cannot be excluded. For example, activin A, which is released in substantial amounts from epicardial adipose tissue, which is highly related to PAT [15, 47], has also been linked to VSMC proliferation [48]. Notably, activin A release is elevated in epicardial adipose tissue from patients with type 2 diabetes and may thus explain higher proliferation of VSMC treated with CM from PAT of patients with type 2 diabetes and PAT of patients without diabetes compared to SAT of patients without diabetes despite similar VEGF content in these CMs. Differently from PAT-induced VSMC proliferation, VAT-induced proliferation could be explained by higher VEGF content in CM from VAT of obese patients with type 2 diabetes and completely prevented by VEGF neutralization. Therefore, further studies remain required to further detail the impact of PAT-derived adipokines on VSMC proliferation.

In conclusion, our results demonstrate that VEGF is mediating CM-induced proliferation of VSMC. As an adipokine and growth factor that is released in high amounts from VAT of obese patients and PAT of patients with type 2 diabetes compared to SAT of lean and nondiabetic controls, VEGF might be a link between adipose tissue inflammation and abnormal VSMC proliferation.
